# Meeting psychosocial needs to improve health: a prospective cohort study

**DOI:** 10.1186/s12885-020-07022-w

**Published:** 2020-06-05

**Authors:** Austyn Snowden, Jenny Young, Jan Savinc

**Affiliations:** Edinburgh Napier University, Sighthill campus, School Health & Social Care, Edinburgh, EH11 4BN Scotland

**Keywords:** Holistic needs assessment, Health status, EQ-5D, Quality of life, Community, Cancer, Support, Psychosocial

## Abstract

**Background:**

Cancer impacts on patients and their families across a range of different domains. For that reason, optimal cancer care has moved away from a disease-centric focus to a more holistic approach in order to proactively support people with their individual needs and concerns. While international policy clearly advocates this agenda, implementation into routine care is limited. Therefore, relevant interventions that measurably improve patient outcomes are essential to understand if this ideal is to become routine multidisciplinary practice. The aim of this study was to analyse the impact of a proactive, holistic, community-based intervention on health-related quality of life in a cohort of people diagnosed with cancer. Secondary aim was to explore the relationship between changes in health status and: cancer type, cancer stage, number of concerns expressed and change in severity of concerns pre and post intervention.

**Method:**

Prospective observational cohort study. A convenience sample of 437 individuals were referred to the service ‘Improving the Cancer Journey (ICJ) in the UK. Each completed the Euroqol EQ-5D-3 L and visual analogue scale (VAS) and a Holistic Needs Assessment (HNA) during initial visit to the service and again at follow-up review, median 84 days later. Change between scores was tested with paired t-tests and relationships between variables with multiple regression models with heteroscedasticity-consistent standard errors.

**Results:**

Participants were White British with median age between 50 and 64 years. Cancer type and stage were varied. EQ-5D utility scores improved at follow-up by 0.121 [0.0891–0.153], *p* < .001, and VAS scores improved by 7.81 [5.88–9.74], *p* < .001. The strongest predictor of change was a decrease in severity of concerns. Cancer stage ‘palliative care’ contributed to a reduction in health status.

**Conclusion:**

This study is the first to show that a holistic community intervention dedicated to supporting the individual concerns of participants had both a statistically significant and clinically meaningful impact on participants’ health-related quality of life. The mean change in EQ-5D scores was more than the ‘minimally important clinical difference’ described in the literature. This is important because while quality of life has multiple determinants, this study has shown for the first time that it is possible to capture a clinically meaningful improvement as a function of reducing someone’s personally identified concerns.

## Background

Globally, following a cancer diagnosis people report a wide range of needs and concerns [1]. The ideal of modern health and social care is therefore to optimise the skills available from a matching range of multidisciplinary professionals to meet these physical, psychological, social, emotional, financial, practical and spiritual needs, whilst at all times keeping the individual at the centre of decisions [2].

However, evidencing the benefits of holistic approaches to the patient is complex, not least because there are numerous interacting factors that impact on outcomes. For instance, there are different approaches to providing holistic care, including different assessment tools and assessor actions that affect the patient experience [3, 4]. Even using the same assessment, individuals respond in different ways according to the professional undertaking the assessment [5], suggesting that there is no such thing as a ‘value free’ assessment of holistic need. Consequently, while policy has recognised the importance of routine, person-centred, psychosocial care [6], concerns relating to implementation barriers, the lack of clarity on the best way to identify needs and poor evidence of impact prevents widespread uptake [7, 8]. Nevertheless, successful interventions exist. Therefore, the most appropriate learning at present comes from successful examples of care delivery consistent with this holistic agenda.

### The intervention – Improving the cancer journey

**Fig. 1:**
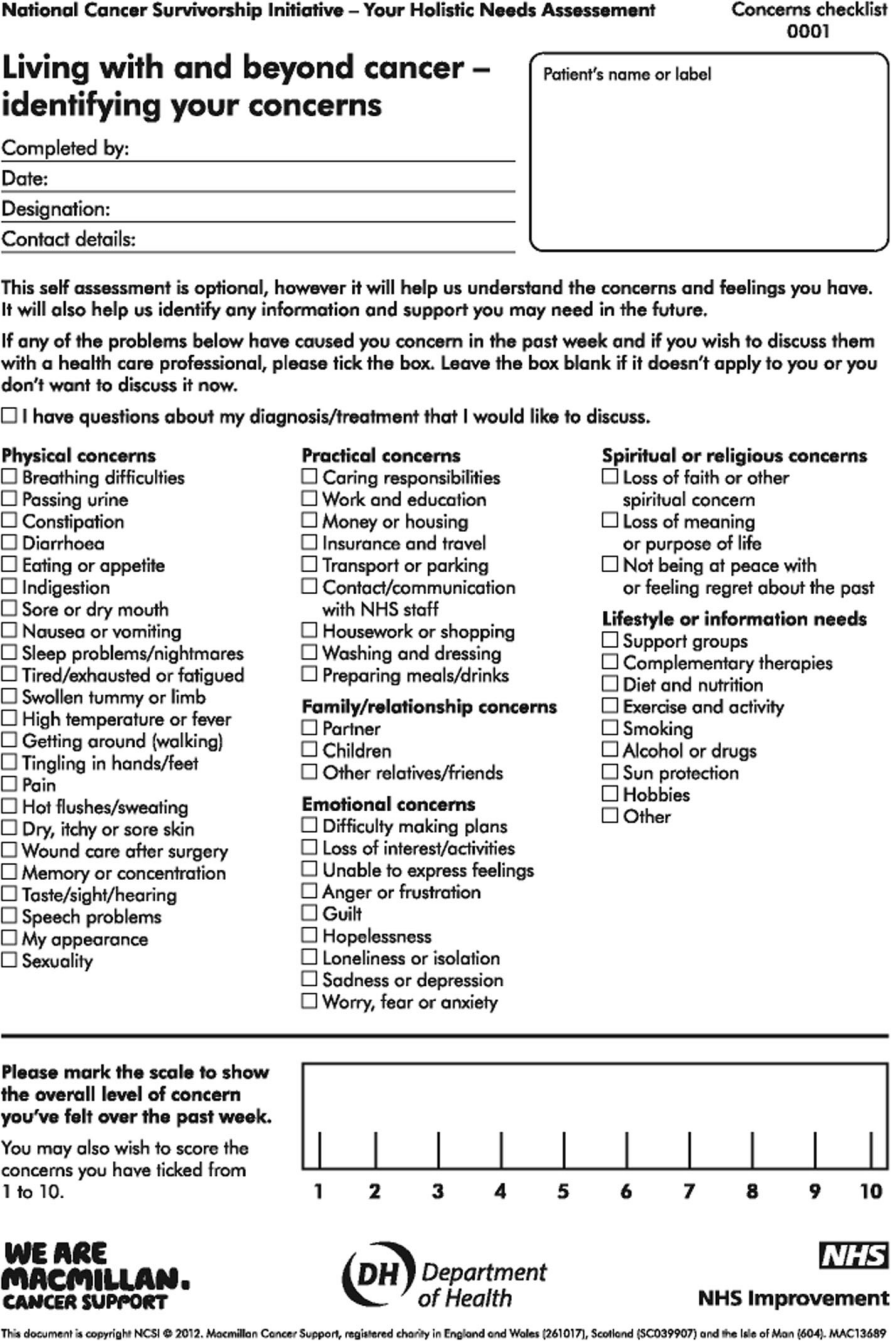
Holistic Needs Assessment

‘Improving the Cancer Journey’ (ICJ) was commissioned in 2014 in Glasgow, Scotland. It is the first community-based cancer service of its kind in the UK and is unique for three interrelated reasons. First, stakeholders are multi-professional. Led by the city council with partners across health, social care, housing and the third sector. Second, the key intervention (Holistic Needs Assessment (HNA) Fig. 1) is proactive: people newly diagnosed with cancer are actively sought out and referred to support. Third, the intervention is coordinated by non-clinical ‘link officers’ rather than health professionals (Table 1).

**Table 1:** The link officer

ICJ link officers are city council employees, not health care professionals. The council currently employ seven link officers. When they first join the service, link-officers have a 3-month induction period where each officer becomes familiar with their role and completes a range of training. Currently all officers are, or are working towards, being accredited with a Level 3 Scottish Vocational Qualification (SVQ) in healthcare support to reflect their competencies in this area. Level 3 SVQ is a vocational qualification academically equivalent to graduate diploma level, or second year of baccalaureate degree.	

In more detail, ICJ writes to every person with a confirmed cancer diagnosis in Glasgow and invites them to access the support, if they wish. At a pre-arranged appointment the link officer meets with their client at a location of their choice. This may be their home, a community venue such as a library or their local hospital (both inpatient and outpatient). During this appointment a HNA (Fig. 1) is carried out, whereby clients are asked to score each of their identified concerns from zero to 10, reflecting the severity of the concern for that person. Based on mutually agreed priorities between the patient and link officer, a care-plan is then co-constructed which details any actions that will be carried out to support the identified concerns. For example, the link officer may provide written information or make a referral to an appropriate agency. The link officer revisits each case; the timing dependant on the clients’ circumstances, severity of concerns raised, care plan details and prognosis. At this review, a second HNA is carried out to identify if the client’s concerns have reduced and/or there are any new concerns. This process continues until the client and the link officer are satisfied that no further support is required.

The service has supported approximately 4000 people since 2014 across a range of cancer types and stages and sociodemographic backgrounds [9]. However, the most common use of the service is from individuals with lung cancer, who are aged between 55 and 64 years and who live in areas of high deprivation, as measured by the Scottish Index of Multiple Deprivation (SIMD). Thirty per cent of ICJ clients were receiving treatment at the time of their first HNA. Most (over 50%) have at least one co-morbidity. The top three concerns for all users of ICJ are financial, fatigue and worry/anxiety. Actions taken by the service include referral to organisations for financial support (including payment of state benefits), referral to other charities for services such as counselling and complementary therapies and referral to social care for assistance with daily living.

There is quantitative and qualitative evidence that this service generates positive outcomes for individuals [9, 10]. Demonstrating a national commitment to this model of care in 2019, Macmillan Cancer Support (a UK charity) and the Scottish Government each pledged £9 million to ensure everyone diagnosed with cancer has a dedicated support worker. According to the Scottish Government [11] this will make Scotland the first country in the UK to offer cancer patients guaranteed emotional, practical and financial advice .

However, despite this public support it remains unclear what, if any, relationship there is between identifying and meeting someone’s personally identified needs and any subsequent impact on self-reported health status. This is important as it would provide currently lacking evidence of effectiveness using standardised measures. In turn, this will improve the ability to generalise findings to other geographical and cancer care settings and lay the foundation for future research to develop a conceptual theory on the relationship between ‘need’ (which may cover a number of domains) and health related quality of life [12].

## Aim

The overarching aim was to analyse the impact of ICJ on self-reported health status using the EQ-5D - 3 L utility measure and visual analogue scale (VAS) [13]. Secondary aim was to explore the relationships between change in health status and cancer type, cancer stage, number of HNA concerns expressed, severity of concerns and change in severity of concerns between pre and post intervention.

### Hypotheses

Primary:

1. There will be a statistically significant difference between EQ-5D scores at baseline and EQ-5D scores post intervention.

Secondary:

2. There will be a relationship between changes in health status and: cancer type, cancer stage, number of concerns expressed and change in severity of concerns pre and post intervention.

## Method

### Design

Prospective observational cohort study.

### Analytic variables

Sociodemographic data included age range, sex, and Scottish Index of Multiple Deprivation (SIMD). These data were collected with consent from the participants who had accessed the intervention at baseline. The following data were collected at baseline and also post intervention: cancer type, cancer stage, and data relating to the HNA process (Fig. 1) including number and mean severity rating of concerns identified. To measure self-reported health status, participants completed the EQ-5D-3 L and Visual Analogue Scale (VAS) at baseline and post intervention. A utility score was computed from the EQ-5D ratings using an algorithm and value sets produced in a UK population study [14] of societal preferences using the Time Trade-off (TTO) method. A utility score of 1 is interpreted as the best possible health, 0 as death, and values of < 0 as being worse than death.

### Participants

In 2018/19 a consecutive, convenience sample of 437 ICJ clients completed the EQ-5D-3 L and VAS on paper versions during their initial visit and again at their follow-up review. Initial assessments were face to face so individuals completed the surveys themselves. Reviews usually occurred over the telephone so the link officer, through conversation, completed it on the participants’ behalf.

### Analytic plan

All data were imported into R (version 3.5.0, using ‘tidyverse’ package version 1.3.0 [15]) and SPSS package for statistics version 23, cleaned and checked for outliers. For the main hypothesis, a paired t-test was run to ascertain the difference in EQ-5D-3 L scores between initial visit and post intervention, and the same for the VAS. For the secondary aim, EQ-5D-3 L and VAS change scores post intervention were tested for associations using univariate linear regressions with sociodemographic, clinical and HNA-related variables, with those found associated entered into two multiple linear regression models to identify likely predictors of change in EQ-5D scores and VAS between assessments. For descriptive statistics, means and confidence intervals were computed for approximately normally distributed variables, proportions for categorical variables, and median and minima and maxima for non-normally distributed variables. Only pairwise complete observations were used in analysis.

Regarding interpretation, the concept of ‘minimally important clinical difference’ (MICD) has been used to explain the amount of change required in a particular test score that represents a clinically meaningful change for the individual taking that test. For example, it has been used to interpret change in measures of asthma control [16] and wellbeing [17], including the EQ. 5D [18], and so this concept was also applied here.

## Results

**Fig. 2:**
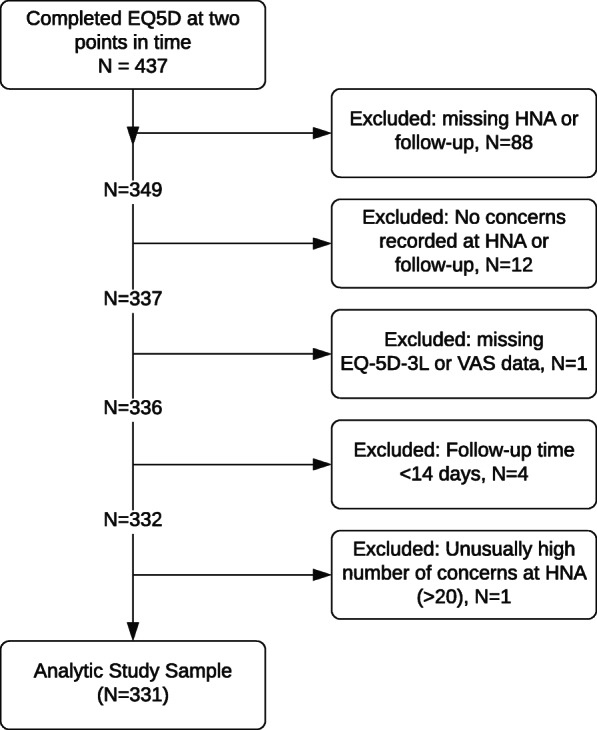
Sample inclusions and exclusions

**Fig. 3:**
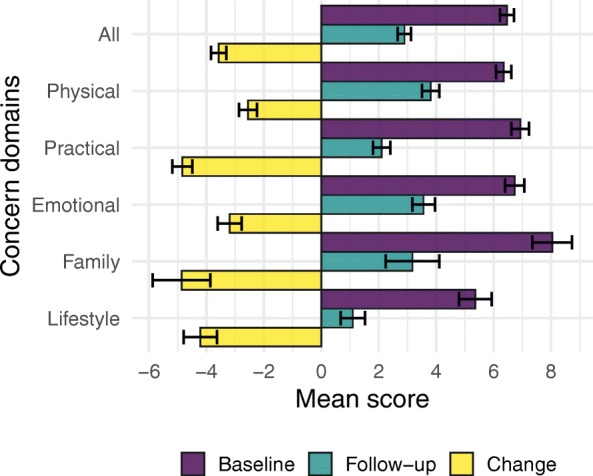
Baseline, Follow-up and Change score for Mean concern severity across domains. Error bars depict 95% CI. The negative change scores correspond to an improvement

HNA data *and* EQ-5D results at baseline and follow-up were obtained for 349 individuals as not every client opts to have a HNA or has a Review (as of August 2019, approximately 6800 clients were referred to ICJ, with approx. 4100 or 60% of referrals completing a HNA, and approx. 1800 or 43% of HNAs also receiving a follow-up HNA). As detailed in Fig. 2, twelve participants were excluded for not having had any concerns recorded at HNA or review, four participants were removed for having baseline and follow-up scores recorded less than 14 days apart, one participant was removed for reporting an unusually large number of concerns in their HNA, and one participant was removed for having an incomplete EQ. 5D. A total of 331 individuals were analysed. The time between assessments ranged from 14 to 456 days, averaging 117 days (median 84). Between baseline and follow up, self reported severity of concern dropped, in line with previous findings [9]. Figure 3 shows the mean change in the different domains of the HNA. There is further detail in supplementary file 1.

Table 2 shows patient characteristics. In summary, the majority of participants were aged between 50 and 64 years, 59% were female, most resided in areas of high deprivation and cancer type and stage were varied. The variable ‘Palliative care’ denotes individuals who identified as receiving palliative care at baseline or follow-up.

**Table 2:** Participant characteristics

*Characteristic*	*Statistic*	*N*
Age, N (%):		330
25 to 49 years	32 (9.70%)	
50 to 64 years	127 (38.5%)	
65 to 74 years	112 (33.9%)	
75 years and over	59 (17.9%)	
Sex, N (%):		325
Female	192 (59.1%)	
Male	133 (40.9%)	
Cancer type, N (%):		331
Bowel	29 (8.76%)	
Breast	71 (21.5%)	
Lung	72 (21.8%)	
Pther	131 (39.6%)	
Prostate	28 (8.46%)	
Cancer stage at baseline, N (%):		273
Living with condition	55 (20.1%)	
Receiving palliative care	26 (9.52%)	
Recently completed treatment (within 1 month)	17 (6.23%)	
Recently diagnosed (1 month)	35 (12.8%)	
Undergoing tests	18 (6.59%)	
Undergoing treatment	122 (44.7%)	
Cancer stage at follow-up, N (%):		322
Living with condition	144 (44.7%)	
Receiving palliative care	55 (17.1%)	
Recently completed treatment (within 1 month)	28 (8.70%)	
Recently diagnosed (1 month)	1 (0.31%)	
Recurrence	1 (0.31%)	
Undergoing tests	9 (2.80%)	
Undergoing treatment	84 (26.1%)	
Palliative care, N (%):		276
Yes	59 (21.4%)	
No	217 (78.6%)	
Deprivation (1 = most deprived), Median [min-max]	3 [1–20]	331

### Primary hypothesis

1. There will be a statistically significant difference between EQ-5D scores at baseline and EQ-5D scores post intervention.

**Fig. 4:**
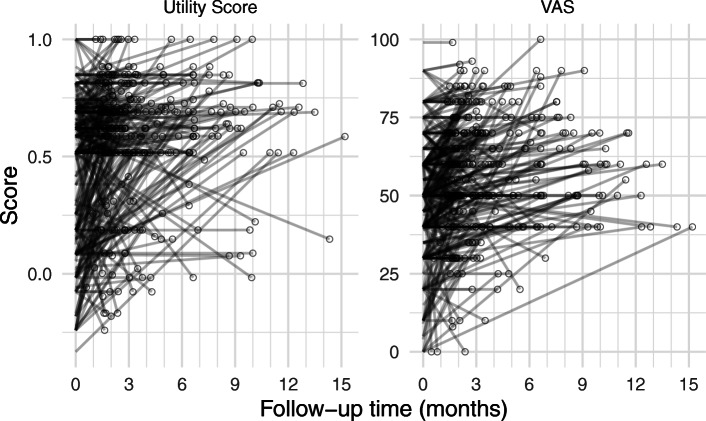
Spaghetti plots showing change of EQ. 5D Utility scores and VAS from baseline to follow-up for each participant. Each partly transparent line segment denotes one participant, with darker lines indicating overlapping trajectories. The follow-up score is marked with a circle for clarity

Table 3 presents the descriptives of the EQ-5D-3 L Utility score and Visual Analogues Scale (VAS) at baseline and follow-up. Figure 4 shows the same data but for each individual participant in spaghetti plots. Both EQ-5D measures increased, indicating an improvement in health status. The distributions of change scores for EQ-5D utility scores and VAS were approximately normal with heavier tails on the positive side, and a large proportion of 0 values. However, because the sample size was sufficiently large, the t-test was assumed to be sufficiently robust to non-normality (Lund & Lund, 2019).

**Table 3:** Descriptive summary of outcomes. The negative difference in concern severity is interpreted as an improvement

*Measure*	*Declined*	*Improved*
Utility score	12.7–17.5%	41.7–48.6%
VAS	6.3–13.6%	30.5–48.0%

Using a paired t-test, the increase in EQ-5D utility scores of 0.121 [0.0891–0.153] at follow-up was found to be statistically significant (*p* < .001), as was the increase in VAS of 7.81 [5.88–9.74] (*p* < .001). Cohen’s d effect sizes were 0.43 [0.27–0.58] for Utility score difference, and 0.42 [0.27–0.58] for VAS, both of which are considered small to moderate. The hypothesis of a significant difference between baseline and follow-up on EQ-5D scores was supported. The mean changes in EQ-5D scores fell within previously published Minimal Clinically Important Difference (MCID) estimates for oncological patients: 0.07 to 0.12 for utility scores [19], and 7 to 12 for VAS [20]. Table 4 shows the estimated proportion of individuals who had a clinically important improvement or decline using the reported MCID values as lower and upper bounds.

**Table 4:** Proportion of individuals whose EQ. 5D scores improved or declined above the MCID threshold, using published lower and upper bound estimates

*Outcome*	*Mean [95% CI]*
Follow-up time (days)	117 [107;126]
Concern severity at baseline	6.47 [6.23;6.71]
Concern severity at follow-up	2.90 [2.66;3.13]
Concern severity difference at follow-up	− 3.57 [− 3.84;-3.30]
VAS at baseline	49.1 [47.1;51.1]
VAS at follow-up	56.9 [54.9;58.9]
VAS difference at follow-up	7.81 [5.88;9.74]
Utility score at baseline	0.45 [0.42;0.49]
Utility score at follow-up	0.58 [0.55;0.60]
Utility score difference at follow-up	0.12 [0.09;0.15]

### Secondary hypothesis

2. There will be a relationship between changes in self-reported health related quality of life and: cancer type, cancer stage, number of concerns expressed, and change in severity of concerns pre and post intervention.

Univariate regressions of EQ-5D scores on age group, gender, cancer type, cancer stage, palliative care, deprivation level, number of concerns reported, follow-up time, and mean change in concerns between assessments can be found in Table 5. Variables that were statistically significantly (*p* < .05) associated with EQ-5D scores were entered into multiple regression models (Table 6). The variables used were: time elapsed between EQ-5D assessments, mean change in concerns between assessments, and palliative care, with the EQ-5D utility score model also using number of concerns as predictor. Utility score differences were only significantly different between 25 and 49 years, and 75 years and over, so Age was not included in the multiple regression.

**Table 5:** Univariate regressions of patient characteristics and outcomes on EQ-5D scores; p-values significant at α < .05 shown in bold. Follow-up time in multiples of 30 day increments was defined as the number of days divided by 30 to approximate number of months

Variable	N	Utility score			VAS		
		Beta	95% CI	***p***-value	Beta	95% CI	***p***-value
Age	330						
25 to 49 years		–	–		–	–	
50 to 64 years		−0.11	−0.22, 0.01	0.068	−4.7	−12, 2.2	0.2
65 to 74 years		−0.11	− 0.22, 0.01	0.067	−5.9	−13, 1.1	0.1
75 years and over		− 0.15	−0.28, − 0.03	**0.019**	− 5.7	− 13, 2.0	0.15
Sex	325						
Female		–	–		–	–	
Male		0.01	−0.05, 0.08	0.7	0.33	−3.6, 4.2	0.9
Cancer type	331						
Bowel		–	–		–	–	
Breast		0	−0.13, 0.12	> 0.9	− 0.99	−8.7, 6.8	0.8
Lung		−0.02	−0.15, 0.11	0.7	−3.5	−11, 4.2	0.4
Other		0.03	−0.08, 0.15	0.6	0.07	−7.1, 7.3	> 0.9
Prostate		0.08	−0.07, 0.23	0.3	1.6	−7.7, 11	0.7
Cancer stage at baseline	273						
Living with condition		–	–		–	–	
Receiving palliative care		−0.08	−0.21, 0.06	0.3	−3.3	−11, 4.9	0.4
Recently completed treatment (within 1 month)		0.04	−0.12, 0.19	0.7	1.3	−8.2, 11	0.8
Recently diagnosed (1 month)		0.08	−0.04, 0.20	0.2	−0.32	−7.7, 7.0	> 0.9
Undergoing tests		0.21	0.05, 0.36	**0.008**	0.76	−8.5, 10	0.9
Undergoing treatment		0.06	−0.04, 0.15	0.2	3	−2.6, 8.5	0.3
Cancer stage at follow-up	322						
Living with condition		–	–		–	–	
Receiving palliative care		−0.08	−0.18, 0.01	0.075	−9	−15, −3.5	**0.001**
Recently completed treatment (within 1 month)		− 0.03	− 0.15, 0.09	0.6	2.2	−4.9, 9.4	0.5
Recently diagnosed (1 month)		−0.13	− 0.72, 0.45	0.6	−9.9	−45, 25	0.6
Recurrence		0.45	−0.13, 1.0	0.13	−4.9	− 40, 30	0.8
Undergoing tests		0.05	−0.15, 0.25	0.6	−11	−23, 1.2	0.077
Undergoing treatment		−0.01	−0.09, 0.07	0.8	−2.8	−7.5, 2.0	0.3
Palliative care	276						
Yes		–	–		–	–	
No		0.09	0.01, 0.18	**0.029**	6.6	1.7, 12	**0.008**
Deprivation (1 = most deprived)	331	0	−0.01, 0.00	0.2	0.06	−0.34, 0.45	0.8
Number of concerns at baseline	331	0.02	0.00, 0.03	**0.025**	0.46	−0.37, 1.3	0.3
Mean change in concern severity at follow-up	331	−0.03	−0.04, − 0.02	**< 0.001**	−1.2	−1.9, − 0.39	**0.003**
Follow-up time (30 day increments)	331	0.02	0.01, 0.03	**0.003**	1.2	0.56, 1.9	**< 0.001**

**Table 6:** Linear multiple regression with White’s heteroscedasticity-consistent standard errors for Utility score change and VAS change at follow-up; *p*-values significant at α < .05 shown in bold

	EQ 5D-3 L Utility value change					EQ 5 D VAS change				
*Predictors*	*Estimates*	*std. Beta*	*CI*	*standardized CI*	*p*	*Estimates*	*std. Beta*	*CI*	*standardized CI*	*p*
Intercept	−0.109		−0.195 – − 0.022		**0.014**	0.459		−3.370 – 4.287		0.814
Time elapsed (30 day increment)	0.012	0.102	−0.007 – 0.030	−0.011 – 0.216	0.207	1.076	0.157	0.122–2.030	0.040–0.274	**0.027**
Mean concern change	−0.04	−0.343	− 0.055 – − 0.024	−0.456 – − 0.230	**< 0.001**	−1.132	−0.166	−2.155 – − 0.109	−0.284 – − 0.048	**0.03**
Number of concerns at baseline	0.016	0.129	0.001–0.032	0.014–0.244	**0.036**					
Palliative care	−0.12	− 0.408	−0.206 – − 0.033	−0.283 – − 0.052	**0.007**	−8.636	−0.501	−14.178 – − 3.095	−0.322 – − 0.090	**0.002**
Observations	276					276				
R^2^ / R^2^ adjusted	0.170 / 0.158					0.087 / 0.077				

Both the EQ-5D utility score and VAS models were heteroscedastic so White’s heteroscedasticity-consistent standard errors were used (HC0, using R ‘sandwich’ package version 2.5–1) [21, 22]. Following assumption testing [23], the omnibus test of the EQ-5D utility score model was significant at F (4,271) = 13.9, *p* < .001, adj. R^2^ = .158, with regression terms *Mean change in concern severity between assessments* significant at *p* < .001, *Palliative care* significant at *p* < .01, and *Number of concerns* significant at *p* < .05. *Time elapsed between assessments* was not a significant predictor. The omnibus test of the VAS score model was significant at F (3,272) = 8.6, *p* < .001, adj. R^2^ = .076, with regression terms *Time elapsed between assessments*, *Mean change in concern severity between assessments* significant at *p* < .001, and *Palliative care* statistically significant at *p* < .0001. Regression coefficients, robust standard errors and confidence intervals for both models can be found in Table 5.

HNA average score decreased, indicating a reduction in severity of concerns (Fig. 3). The mean concern severity was 6.47 [6.23–6.71] at baseline, dropping to 2.90 [2.66–3.13] post intervention. Only three individuals (< 1%) showed increase in severity of concern post intervention. Mean concern severity was independent of the number of concerns (Spearman’s ρ = .076, *p* = .17). In the EQ-5D utility score change model, the strongest predictor was *Mean concern change* (β = − 0.34), meaning that a one standard deviation (1SD) decrease in concern severity at follow-up corresponded to a 0.34SD increase in utility score. Next strongest predictor was *Palliative care*, which contributed − 0.408SD to the EQ-5D utility score change. Finally, when the *number of concerns* increased by 1SD, the utility score increased by 0.13SD. The time elapsed between EQ-5D assessments was not a significant predictor in the model.

In the VAS model, the strongest predictor was *Palliative care*, which contributed approximately − 8 points on the VAS scale, followed by *Mean concern change*, where a 1SD decrease in concerns corresponded to a 0.17SD increase in VAS. *Time elapsed between assessments* was a significant predictor of VAS change in the model, corresponding to a 0.16SD increase in VAS in a 1SD time increase.

## Discussion

This study has described a significant association between change in HNA score and self-reported health status. Following intervention from ICJ, mean HNA concern severity reduced from 6.4 [6.23–6.71] to 2.9 [2.66–3.13], consistent with the decrease seen in the wider ICJ population [9]. Concurrently, EQ-5D score increased from 0.45 [0.422–0.488] to 0.57 [0.547–0.604], while VAS scores increased from 49 [47.1–51.1] to 57 [54.9–58.9]. This EQ-5D utility score difference of 0.12 [0.0891–0.153] and VAS difference of 7.81 [5.88–9.74] are considered to be above the ‘minimally important clinical difference’ (MICD) in EQ-5D scores described by Coretti et al., [19], and Pickard et al. [24]. In other words, this level of improvement has been described as an important and meaningful improvement for patients [25]. This is also consistent with qualitative evidence [10] on the perceived benefits of using ICJ.

To further contextualise the scores in this study, Supplementary file 2 presents mean baseline and post intervention EQ-5D utility scores from participants in this study, according to cancer type. The same table also contains a reference range of the highest and lowest mean EQ-5D utility scores for the same cancer types, obtained from international studies specifically designed to ascertain EQ-5D population norms. These values show that the ICJ cohort recorded some of the lowest quality of life scores published in the cancer literature. The intervention is therefore not just clinically meaningful but also successfully reaching the population that requires it the most.

Overall, the models explained a moderate to small amount of variance (approximately 16% for utility scores, and 8% for VAS). The strongest predictor was ‘mean concern change’. Over the same period of time that the EQ-5D scores increased, the HNA mean level of concern severity decreased. Receiving palliative care and the number of concerns were also significant predictors, along with time between assessments on the VAS scores, but not the index scores. However, a proportion of the improvement remains unexplained. There is a missing explanatory variable, consistent with the interpretation that the *process of ICJ* is also contributing to the change in health status. For example, identifying a larger number of concerns at baseline was associated with increased health status at follow up. This also points to the process of ICJ being a determinant of improvement: identifying more concerns leads to more engagement with the services on offer, resulting in better outcomes. However, this remains unknown at present because there is no measure of impact of specific services. This hypothesis will be explored in future research by recording attendance and satisfaction ratings of all the services provided and signposted by ICJ.

Previous research investigating the association between needs assessment and improved outcomes has predominantly focused on measuring impact through a range of measurable outcomes such as distress, anxiety, depression and pain using specific tools such as the Distress Thermometer (DT) [26–28]. Qualitative evidence on the use of HNA has shown that it can improve communication between patients and clinicians, providing an opportunity to discuss non-clinical concerns and signpost patients to a variety of different services [29, 30]. However, assessment alone does not always lead to improved outcomes. Sandsund et al., [29] did not find a statistically significant difference in quality of life after using the HNA in 124 women diagnosed with gynaecological cancer. Hollingworth et al. [31] found no evidence of an effect on distress or quality of life, and concluded that the timing of the assessment and the profession of the assessor can impact on outcomes.

The HNA assessor in ICJ was a *non-clinical* expert. Link officers come to this role with backgrounds in financial inclusion and city council processes, and then undertake a three-month training programme to become specialists. They are therefore equipped with a range of skills and knowledge to help navigate people affected by cancer through the complex systems within health but especially through social care and the third sector. In other studies only limited training was provided to the assessors [7]. This is likely to impact on the quality of the HNA interaction and the knowledge and confidence required to make referrals across different services and sectors. Further, it is rational to suggest that people tailor responses to what they perceive to be the expertise of the person conducting the consultation [32]. Accordingly, in this study participants commonly identified non-clinical concerns such as finances and worry/anxiety. These concerns have been identified as being a substantial burden with individuals much more likely to rate their physical health, mental health, and satisfaction with social activities and relationships as poor compared to those with no financial hardship [33]. For that reason, relieving financial burden is likely to have had a substantially positive impact on other areas of concern, which may also add to the interpretation of the findings in this study.

Identifying and assessing individual concerns [34–36] is unarguably beneficial as it can help, amongst other things, with resource allocation. However, to our knowledge, this is the first study to quantify what this means to individuals’ health related quality of life. This is important because while quality of life has multiple determinants this study has reported that it is possible to capture a meaningful improvement in quality of life as a function of reducing someone’s personally identified concerns.

### Strengths and limitations

The current study has several strengths. This is the first examination of health status over time in a large and heterogeneous sample of cancer patients who have all been supported through the HNA process. The primary limitation is that the sample was not random, and the time between EQ-5D assessments was not standardized. Some degree of improvement over time was anticipated. The way ICJ functions is that assessments are followed by referrals and then followed by further ICJ contact. Therefore, over this time period it is likely that individuals may, for example, finish their treatment and report a higher health status. However, the time elapsed between assessments was only a significant predictor of improvement in VAS but *not* utility scores. Nevertheless, as stated, most of the improvement was unexplained. While a broad array of variables were considered for the model we acknowledge that other variables may have influenced the findings. For example, information on comorbidities and more detailed information on participant’s use of other services and interactions with other professionals would have been beneficial. Future research should identify a broader array of variables including sociodemographic, interpersonal (patient and assessor interaction) and clinical to explore the relationship between needs assessment and health related outcomes.

## Conclusion

The primary aim of this research was to document any changes in self-reported health status following intervention from a cancer service. Health status significantly increased following intervention from ICJ. This is noteworthy because at a time where the cancer workforce is stretched and patient numbers are increasing there is an urgent need to rethink how to use resources efficiently without negatively impacting on patient care. The fact that the assessors in this study were non-health based could well be a model to follow – primarily due to their expertise and the types of concerns they tended to elicit and manage. This sample had complex needs with a large proportion residing in areas of high deprivation, with a poor cancer prognosis and with baseline levels of health status that were considerably lower than other cancer populations. That they can be helped in a clinically meaningful way bodes well for those needing similarly targeted support in the future. These results encourage wide application of HNA and supportive care planning combined with approaches like ICJ that tailor support based on need.

## Supplementary information

**Additional file 1: Supplementary file 1**. Changes over concern domains. Table reporting mean changes in HNA concern severity across physical, practical, emotional, family and lifestyle domains.

**Additional file 2: Supplementary file 2**. EQ-5D utility scores from relevant publications. Table of the highest and lowest mean EQ-5D utility scores for the same cancer types as our sample, obtained from international studies specifically designed to ascertain EQ-5D population norms on these cancer types.

## Data Availability

The datasets used and/or analysed during the current study are available from the corresponding author on reasonable request.

## Abbreviations

HNA: Holistic Needs Assessment
ICJ: Improving the Cancer Journey
EQ-5D: EuroQol 5 Dimension
VAS: Visual Analogue Scale
