# Supplementary material for: Meeting psychosocial needs to improve health: a prospective cohort study
Source: BMC Cancer. 2020 Jun 5;20:528. doi: 10.1186/s12885-020-07022-w (PMC7275579; doi:10.1186/s12885-020-07022-w)
Supplement: Supplementary file 1 — Additional file 1: Supplementary file 1. Changes over concern domains. Table reporting mean changes in HNA concern severity across physical, practical, emotional, family and lifestyle domains. [file 12885_2020_7022_MOESM1_ESM.docx]

**Changes over concern domains**

The Spiritual needs domain had very few reported concerns and was omitted from the analysis. Mean concern severity improved at Follow-up for all Domains (see Supplementary Table 1). The largest improvements were observed in the Family & relationship and Practical concern domains. The Family domain was also most severe at baseline. The Practical domain improvement is the more significant with 76% of ICJ clients reporting concerns in that domain compared to 10 in the Family & relationship domain. This is consistent with previous ICJ results^[[1]](#footnote-1)^ of *Money & housing* being the most commonly reported concern by a large margin, though not necessarily the most severe, and the most severe concerns being *Children*, *Partner*, and *Other relatives or friends.* The Lifestyle Domain was least severe at follow-up but was also least severe at baseline.

The smallest improvement was in the Physical domain which was the second most commonly reported. We suspect the relatively low improvement is because ICJ is primarily a non-clinical service with the range of available services varying across concern Domains. Some needs are well-supported by a range of services, whereas for others the range is limited to referrals to clinical specialists or self-help resources. Overall, improvements were observed in all domains, however further research is warranted into the services referred to and their outcomes.

| *Domain* | *Measure* | *N* | *Proportion reported* | *Mean* | *CI* |
| --- | --- | --- | --- | --- | --- |
| All | Baseline | 331 | 100% | 6.47 | [6.23 6.71] |
| All | Follow-up | 331 | 100% | 2.90 | [2.66 3.13] |
| All | Change | 331 | 100% | -3.57 | [-3.84 -3.3] |
| Physical | Baseline | 246 | 74% | 6.35 | [6.09 6.62] |
| Physical | Follow-up | 246 | 74% | 3.81 | [3.51 4.11] |
| Physical | Change | 246 | 74% | -2.55 | [-2.87 -2.23] |
| Practical | Baseline | 252 | 76% | 6.93 | [6.62 7.24] |
| Practical | Follow-up | 250 | 76% | 2.10 | [1.8 2.4] |
| Practical | Change | 250 | 76% | -4.84 | [-5.19 -4.49] |
| Emotional | Baseline | 153 | 46% | 6.73 | [6.4 7.06] |
| Emotional | Follow-up | 151 | 46% | 3.56 | [3.16 3.95] |
| Emotional | Change | 151 | 46% | -3.19 | [-3.61 -2.77] |
| Family | Baseline | 32 | 10% | 8.04 | [7.35 8.73] |
| Family | Follow-up | 32 | 10% | 3.18 | [2.24 4.11] |
| Family | Change | 32 | 10% | -4.86 | [-5.87 -3.86] |
| Lifestyle | Baseline | 93 | 28% | 5.36 | [4.79 5.93] |
| Lifestyle | Follow-up | 92 | 28% | 1.10 | [0.679 1.52] |
| Lifestyle | Change | 92 | 28% | -4.21 | [-4.8 -3.63] |

Supplementary Table 1. Descriptive summary of Baseline, Follow-up and Change score for Mean concern severity across domains.

1. See references in main paper. There are further reports online also: Snowden A, Young J: *Evaluation of the Glasgow RTS Programme: Improving the Cancer Journey*. London; 2018. ﻿<https://www.macmillan.org.uk/_images/Glasgow-improving-cancer-journey-programme-full-evaluation-2017_tcm9-324593.pdf>

   Snowden A, Young J: *Evaluation of Improving Cancer Journeys*. London; 2016. ﻿<http://www.macmillan.org.uk/about-us/what-we-do/evidence/research-publications/research-and-evaluation-reports> [↑](#footnote-ref-1)
