# Supplementary material for: Meeting psychosocial needs to improve health: a prospective cohort study
Source: BMC Cancer. 2020 Jun 5;20:528. doi: 10.1186/s12885-020-07022-w (PMC7275579; doi:10.1186/s12885-020-07022-w)
Supplement: Supplementary file 2 — Additional file 2: Supplementary file 2. EQ-5D utility scores from relevant publications. Table of the highest and lowest mean EQ-5D utility scores for the same cancer types as our sample, obtained from international studies specifically designed to ascertain EQ-5D population norms on these cancer types. [file 12885_2020_7022_MOESM2_ESM.docx]

| *Summary of the aims of key studies* | *Cancer type* | *Published EQ5D utility score range* | *Notes* | *Observed*  *EQ5D utility score in study (baseline – follow-up)* |
| --- | --- | --- | --- | --- |
| To create adjusted EQ5D scores for a range of difference cancer types in UK (Pickard et al., 2016).    Describe different EQ5D scores associated with different treatment stages (Lidgren, Wilking, Jönsson, & Rehnberg, 2007).  Systematic review of 57 papers examining range of EQ5D scores in a wide range of cancer types (Pickard et al, 2007)  Examine impact of various treatments on metastatic breast cancer, measured with EQ5D (Shiroiwa et al., 2017*;* Färkkilä et al., 2014; Pottel et al., 2015)  To assess the validity of health utility measures in Head and Neck Cancers (Noel et al., 2015)  Systematic review of health state utility values in metastatic lung cancer with a focus on previously treated patients (Paracha et al, 2018) | Breast | 0.45-0.84 | Lower value is metastatic disease. Non-UK studies used UK TTO values | 0.50-0.61 |
|  | Colorectal | 0.41-0.91 |  | 0.48-0.51 |
|  | Head/neck | 0.62-0.83 | Low score represents pre-therapy measure – high is mid therapy | 0.51-0.64 |
|  | Lung | 0.19-0.91 | <30 days from death to more than year away (Korean study) | 0.44-0.53 |
|  | Prostate | 0.51-0.90 |  | 0.49-0.67 |

To further contextualise the scores in this study, the table above shows mean baseline and post intervention EQ-5D utility scores from participants in this study, according to cancer type. The same table also contains a reference range of the highest and lowest mean EQ-5D utility scores for the same cancer types, obtained from international studies specifically designed to ascertain EQ-5D population norms on these cancer types. Figures state that the ICJ cohort recorded some of the very lowest quality of life scores published in the cancer literature. The intervention is therefore not just clinically meaningful, but also successfully reaching the population that requires it the most.
